# Single-Drug Approach with Edoxaban is Effective for Resolving Non-Acute Cancer-Associated Venous Thrombosis: A Single-Arm Retrospective Analysis

**DOI:** 10.3390/cancers12071711

**Published:** 2020-06-28

**Authors:** Hirokazu Toshima, Atsushi Hisamatsu, Kouji Kobayashi, Hiroo Ishida, Ken Shimada

**Affiliations:** 1 Medical Oncology, Showa University Koto Toyosu Hospital, 5-1-38 Toyosu, Koto-ku, Tokyo 135-8577, Japan; hisamatsuatsushi@gmail.com (A.H.); koujikoujikouji@live.jp (K.K.); hishida@med.showa-u.ac.jp (H.I.); shimakenken60@hotmail.com (K.S.); 2 Medical Oncology, Showa University Northern Yokohama Hospital, 35-1 Chigasaki-chuo, Tsuzuki-ku Yokohama-shi, Kanagawa 224-8503, Japan

**Keywords:** edoxaban, DOAC, single-drug approach, cancer-associated venous thrombosis, deep-vein thrombosis, pulmonary embolism, venous thromboembolism, D-dimer

## Abstract

Recently, cancer-related venous thromboembolism (VTE) has been termed “cancer-associated thrombosis (CAT)” and is the focus of current research. We retrospectively investigated the efficacy of a single-drug approach with edoxaban for the treatment of non-acute CAT. Thirty-two non-acute CAT patients who received edoxaban were analyzed. The primary endpoint of this analysis was the thrombus disappearance rate at the first evaluation. Secondary endpoints included progression/recurrence of VTE, major bleeding, and D-dimer levels. The thrombus disappearance rate was 62.5%. Therefore, the null hypothesis for the primary endpoint (thrombus disappearance rate of ≤32.0%) was rejected (*p* = 0.00038) based on the rate of the previous study as the historical control. Recurrent VTE and major bleeding occurred in two patients each. After the start of treatment with edoxaban, a significant difference in D-dimer levels was observed (*p* = 0.00655). We demonstrated that a single-drug approach with edoxaban is a potential treatment option for non-acute CAT.

## 1. Introduction

The Japan VTE Treatment Registry, a multicenter cohort study of patients with venous thromboembolism (VTE) in Japan, has shown that cancer is the biggest risk factor for VTE [1]. The incidence of VTE among patients with cancer is increasing annually [2] and is four to eight times higher than in patients without cancer [3,4,5]. Moreover, the increased risk of VTE is thought to be related to cancer treatment. For example, surgery for cancer increases the risk of fatal pulmonary embolism (PE) and chemotherapy can cause VTE [6,7,8]. VTE is the leading cause of non-cancer-related death in patients with cancer [9]; thus, caution should be exercised concerning VTE. Recently, cancer-related VTE has been termed “cancer-associated thrombosis (CAT)” and has become the focus of current research. In this study, we focused on VTE and referred specifically to cancer-associated venous thrombosis as CAT.

There are various guidelines for the treatment of VTE. Until recently, there has been little evidence supporting the efficacy of direct oral anticoagulants (DOACs) for the treatment of CAT. The 2016 American College of Chest Physicians guideline recommends low-molecular-weight heparin (LMWH) as a treatment for acute CAT [10]. However, as the noninferiority of edoxaban to LMWH has been proved in the 2017 Hokusai VTE cancer study [11], evidence for the use of DOACs to treat CAT has been increasing gradually. The revised 2019 American Society of Clinical Oncology guidelines added DOACs to the list of treatments for CAT in addition to LMWH [12].

Treatment with edoxaban for acute VTE requires prior heparin administration. However, rivaroxaban and apixaban can be used directly as monotherapy for the treatment of acute VTE. This single-drug approach that uses a single drug from the start of treatment to maintenance therapy is simple and useful for outpatient treatment. Prior heparin administration is not always necessary for the treatment of non-acute VTE with edoxaban but there is little evidence of a single-drug approach to treat CAT. Therefore, we retrospectively investigated the efficacy of edoxaban single-drug approach for the treatment of non-acute CAT.

## 2. Materials and Methods

### 2.1. Clinical Background of This Study

D-dimer is a widely studied biomarker in the diagnosis and management of VTE [13,14]. In the Vienna modification of the Khorana score, D-dimer has been added as a biomarker of CAT prediction. It has also been reported that D-dimer levels show an association with the disease state, prognosis, and the risk of VTE not only at diagnosis but also during chemotherapy [15,16]. More recently, a clinical-prediction model that uses only two variables including D-dimer was proposed [12,17,18]. Measuring D-dimer levels is drawing increasing attention for monitoring VTE. In our clinical practice, D-dimer levels had been routinely measured once a month during chemotherapy treatment. Using this clinical background data, our study retrospectively reviewed these cancer patients’ medical history relevant to VTE.

An automated coagulation analyzer (Coapresta 2000; Sekisui Medical Co., Ltd., Tokyo, Japan) was used to analyze all specimens. Nanopia D-dimer (Sekisui Medical Co., Ltd., Tokyo, Japan) was used as a reagent; the reference range of D-dimer levels was 1.0 μg/mL or less. When D-dimer levels exceeded this reference range, contrast-enhanced computed tomography (CT) was performed to exclude PE and deep-vein thrombosis (DVT) applying a VTE protocol (pulmonary-arterial and lower-extremity-venous phase). In addition to CAT that lasted more than 14 days from the onset [19], we also defined a diagnosed asymptomatic CAT as non-acute CAT.

### 2.2. Patients

**Figure 1:**
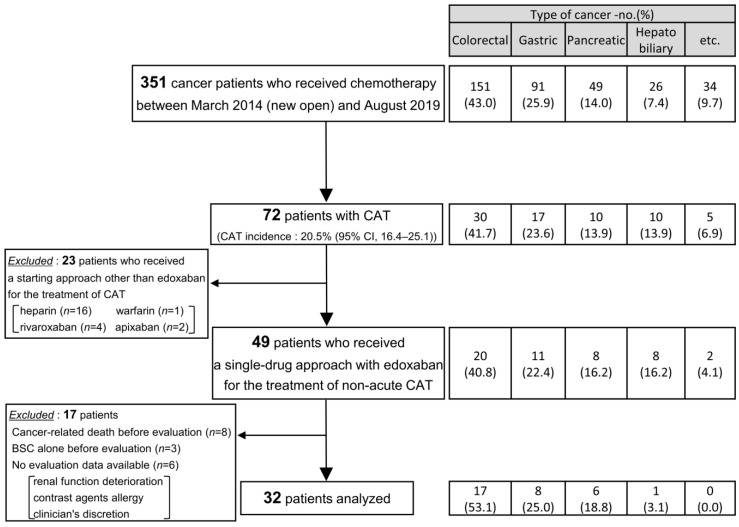
Flowchart and table indicating the number of patients included in and excluded from this analysis.

We retrospectively reviewed data on 351 cancer patients who initiated chemotherapy between March 2014 and August 2019 at the Department of Medical Oncology, Showa University Koto Toyosu Hospital, and identified 49 non-acute CAT patients who received a single-drug approach with edoxaban. Of these, 32 subjects were available for follow-up evaluation and were analyzed. Figure 1 shows the patients who were excluded from this study.

The study was approved by the Institutional Review Board of Showa University Koto Toyosu Hospital (permission number: 19T7039). The study was conducted in accordance with the Helsinki Declaration guidelines. This retrospective study was conducted using our clinical database. All patient data were protected and kept confidential. During data acquisition, personal information that could be used to identify patients was eliminated. Through the homepage of the hospital, patients were informed about the purpose of the study and provided with the option to opt-out; hence, no additional patient consent was required.

### 2.3. Treatment

Edoxaban was administered orally at a fixed dose of 60 mg once daily without prior administration of heparin. It was administered at a lower dose (30 mg once daily) in patients with a creatinine clearance of 30 to 50 mL/min or a body weight of 60 kg or less. None of the patients received concomitant treatment with potent P-glycoprotein inhibitors.

### 2.4. Evaluations

All patients were followed up for 12 months after treatment (390 days) or until death. In this study, 0 months indicated 0–30 days, 1 month indicated 31–60 days, and 2 months indicated 61–90 days periods. The primary endpoint of this analysis was the thrombus disappearance rate at the time of the first evaluation performed within 3 months from the start of treatment. Secondary endpoints included progression/recurrence of VTE, major bleeding, and D-dimer levels. We performed contrast-enhanced CT scans every 2–3 months to determine the tumor reduction effect. Simultaneously, the therapeutic effect on thrombus was also evaluated. Referring to the RECIST guidelines (version 1.1) [20], the following evaluation methods were defined to quantitatively evaluate the therapeutic effect of edoxaban on the thrombus. First, the size of the thrombus at baseline was evaluated and used as a control for future measurements. If multiple thrombi were observed during the baseline evaluation, up to five representative thrombi were selected as the target. Next, the sum of the major axes of all target thrombi was calculated as the sum of the baseline diameters. The effect was determined by comparing the sum of the baseline diameters; shrinkage was determined when the sum of the major axes of the target thrombi was reduced by 30% or more, and progression was determined when there was an increase of 20% or more. According to the International Society on Thrombosis and Haemostasis criteria, major bleeding was defined as a reduction in hemoglobin levels of 2 g/dL or more, bleeding that required more than 2 units of blood transfusion, or bleeding or fatal bleeding in important areas [21].

### 2.5. Statistical Analysis

The binomial test was used to evaluate the thrombus disappearance rate as the primary endpoint. A previous study reported that the thrombus disappearance rate at the end of the scheduled treatment period was 31.6% and 62.0% for heparin/warfarin and rivaroxaban, respectively [22]. Based on this historical control data, we defined the threshold of thrombus disappearance rate at the first evaluation as 32.0% and the expected rate as 62.0%. It was estimated that sample size of at least 22 patients was required to allow a one-tailed significance level of 2.5% and a power of 80% using the binomial test. In our retrospective study, 32 patients were included in the analysis, which met the sample size requirements. The post-hoc power in this case was 91.4%. The Friedman test was used to distinguish between the baseline and the subsequent D-dimer data as the secondary endpoint. Statistical analyses were performed using R version 3.5.2 (Foundation for Statistical Computing, Vienna, Austria) and SPSS Statistics version 19.0 (IBM Corp., Armonk, NY, USA).

## 3. Results

Data are presented as median (interquartile range (IQR)). The median age was 68 years (IQR, 60.75–75.25). The median period of follow-up at the time of analysis (August 2019) for the 32 patients was 335.5 days (IQR, 245–390). The most common primary site was colorectal cancer. Twenty-five patients received low doses of edoxaban (30 mg, once daily). The characteristics of the 32 eligible patients are listed in Table 1.

### 3.1. Primary Endpoint

**Figure 2:**
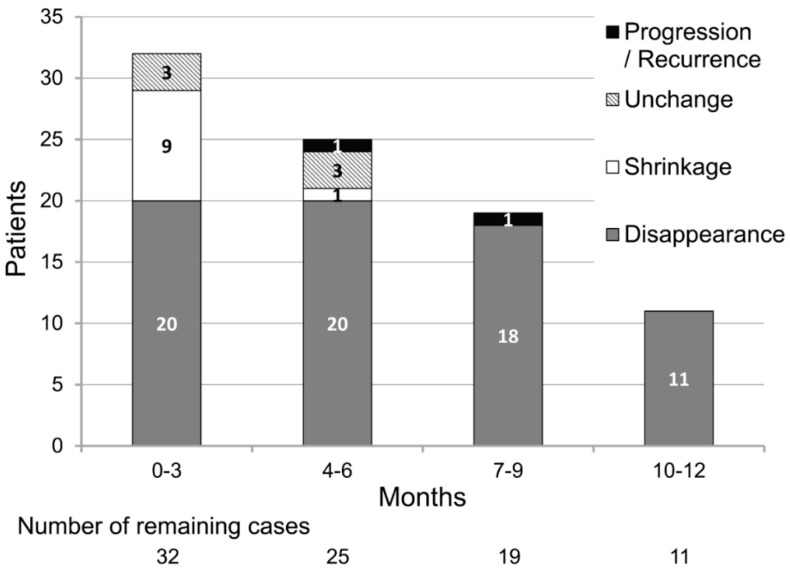
Results of repeat venous thromboembolism (VTE) evaluation during the overall analysis period.

The median number of days from the start of treatment to the first evaluation was 60.5 (IQR, 45.75–68.25). At the first evaluation, the thrombus disappeared in 20 patients; thus, the thrombus disappearance rate was 62.5% (95% confidence interval (CI), 43.7–78.9) (Figure 2, Table 2).

Therefore, the null hypothesis for the primary endpoint (thrombus disappearance rate of ≤ 32.0%) was rejected (one-tailed, *p* < 0.025 regarded as significant, *p* = 0.00038). In the analysis of the low-dose edoxaban (30 mg once daily) group only (*n* = 25), the thrombus disappeared in 17 patients; thus, the thrombus disappearance rate was 68.0% (95% CI, 46.5–85.1). Therefore, the null hypothesis was rejected (one-tailed, *p* < 0.025 regarded as significant, *p* = 0.00024) even for the low-dose edoxaban group.

**Figure 3:**
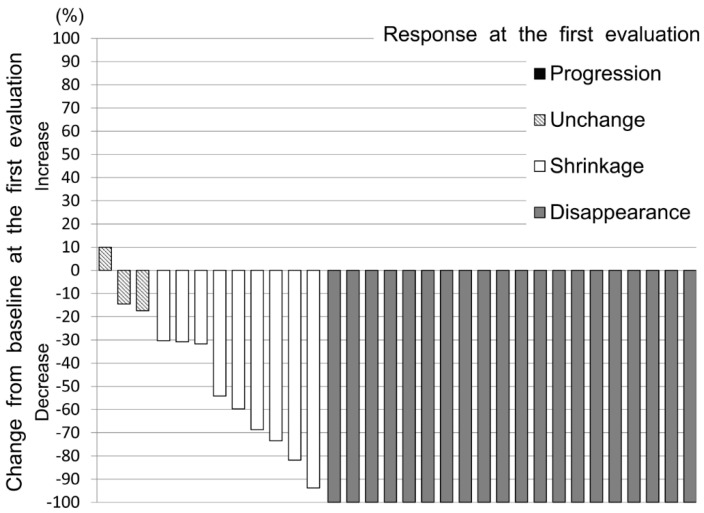
Waterfall plot of confirmed change from baseline at the first evaluation.

**Figure 4:**
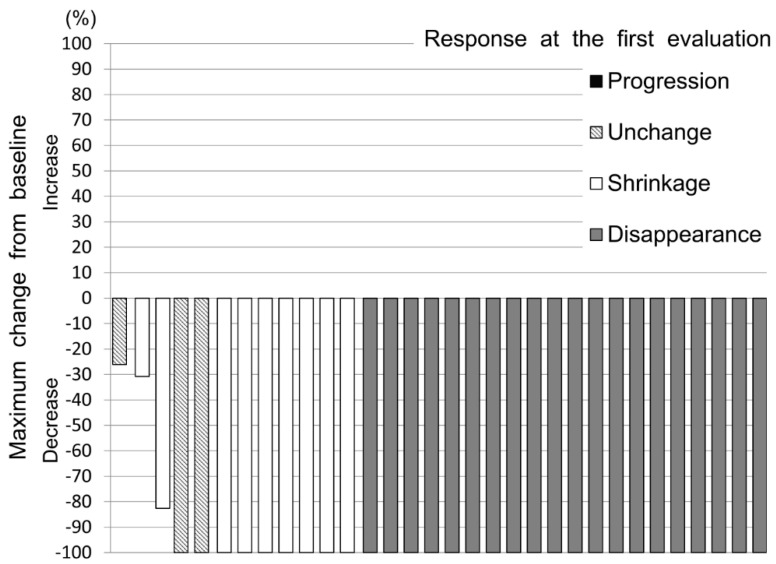
Waterfall plot of confirmed maximum change from baseline.

None of the patients experienced progression of their VTE at the first evaluation; thus, the disease-control rate was 100%. A waterfall plot of the change from the baseline at the first evaluation is shown in Figure 3, and a plot of the maximum change from the baseline is shown in Figure 4.

The bars are color-coded according to the response at the first evaluation. The median number of days from the start of treatment to the maximum change was 72.5 (IQR, 51.5–111.75). At the time of maximum change, the thrombus disappeared in 29 patients, and the final thrombus disappearance rate was 90.6% (95% CI, 75.0–98.0).

### 3.2. Secondary Endpoints

**Figure 5:**
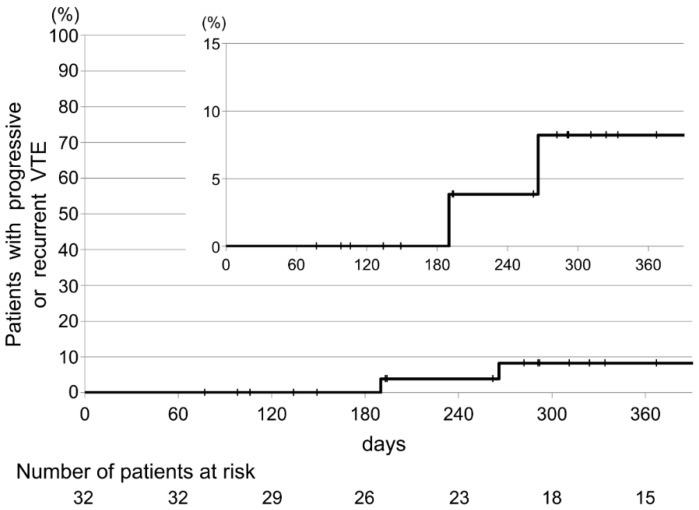
Kaplan–Meier cumulative-event rates for progressive or recurrent venous thromboembolism.

**Figure 6:**
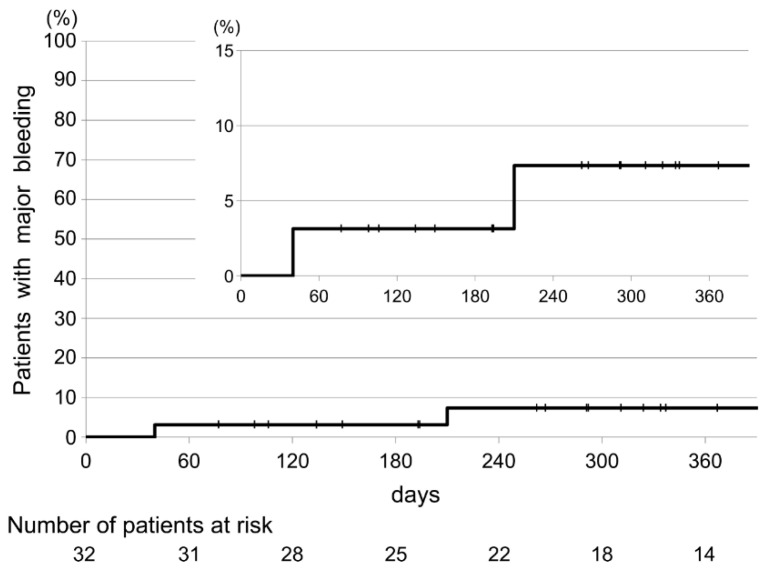
Kaplan–Meier cumulative-event rates for major bleeding.

The secondary endpoints are shown in Table 2. Recurrent VTE occurred in two patients (6.25% (95% CI, 0.8–20.8)). Major bleeding also occurred in two patients (6.25% (95% CI, 0.8–20.8)). The source of bleeding was primary gastric cancer and bladder invasion from gastric cancer with peritoneal dissemination. Both patients required blood transfusions but without clinical urgency. The time to the occurrence of progressive or recurrent VTE and major bleeding is shown in Figure 5 and Figure 6.

**Figure 7:**
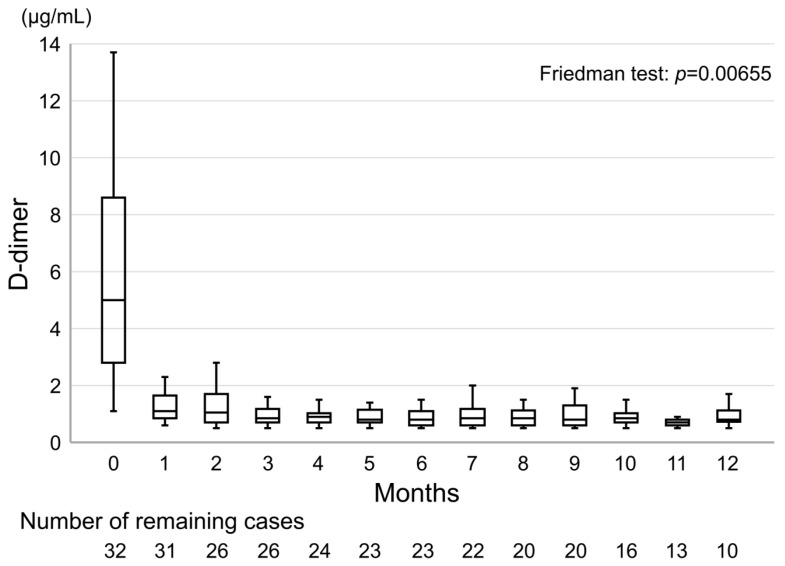
The transition of d-dimer levels during the overall analysis period.

The median D-dimer level at the time of diagnosis was 5.0 μg/mL (IQR, 2.8–8.6) and median D-dimer level at 1 month after the start of treatment with edoxaban was 1.1 μg/mL (IQR, 0.85–1.65). Figure 7 shows the transition of D-dimer levels using box-and-whisker plots. After the start of treatment with edoxaban, a significant difference in D-dimer levels was observed (*p* < 0.05, regarded as significant, *p* = 0.00655).

## 4. Discussion

In the single-center retrospective study reported here, we tested the single-drug approach with edoxaban and showed that the thrombus disappearance rate at the first evaluation was 62.5%, thus meeting the study’s primary endpoint. In our analysis, we used the results of the J-EINSTEIN DVT and PE program study as control [22]. In this previous study, data at 3, 6, or 12 months were recorded at the discretion of the attending physicians and showed a median of 6 months, and the study was not limited to patients with an increased coagulation activity owing to cancer. In our study, the primary endpoint was determined from data after 60.5 days (median) and our analysis targeted patients with cancer at a high risk of VTE. We could reach the projected thrombus disappearance rate (62.5%). Recurrence and major bleeding were within the acceptable range, and the results were similar to those from studies (the Hokusai VTE Cancer study and SELECT-D trial) of patients with cancer [11,23]. Although comparisons with previous studies need to be interpreted with caution because of the differences in study design, our results suggest that a single-drug approach with edoxaban is a potential treatment option for non-acute CAT.

There is still insufficient evidence for which DOACs should be selected to treat a patient. Each DOAC has its characteristics. Drug-drug interactions are an important issue for oncologists; caution should be exercised regarding cytochrome P450 (CYP). CYPs are a family of enzymes involved in drug metabolism, with CYP3A4 being involved in the metabolism of more than 50% of drugs used in clinical practice [24]; it is responsible for the intestinal first-pass effect in the absorption process of oral drugs. Chemotherapy includes oral anticancer drugs, and the use of oral medication increases with supportive care toward the end of life. As more oral drugs are metabolized by CYP3A4, competitive inhibition occurs in the small-intestinal epithelium. This inhibition may increase the blood levels of each drug and the risk of adverse events. Although rivaroxaban and apixaban are substrates of CYP3A4, less than 4% of edoxaban is metabolized by CYP3A4 [25]. The benefit resulting from limited drug-drug interactions of edoxaban makes the drug valuable as a non-acute CAT-treatment option.

In unprovoked VTE, the normalization of D-dimer levels can be used to determine when to end anticoagulation therapy [26,27]. Patients with high D-dimer levels 1 month after discontinuation of anticoagulant therapy are at an increased risk of VTE recurrence [28,29,30]. On the other hand, these were not studies on patients with cancer and indefinite anticoagulation therapy is recommended for CAT [10]. However, unprovoked VTE also occurs in undiagnosed cancers [31], and maintaining the normalization of D-dimer levels after starting anticoagulant therapy is important in CAT. In our study using edoxaban, a significant decrease in D-dimer levels was observed after the start of treatment, and this decrease was maintained for 12 months (Figure 6).

However, this study had several limitations. (1) It was a single-arm retrospective analysis only in Japanese patients with limited sample size. Moreover, there was a problem with exclusion because evaluation data were not available. (2) Biases of the attending physicians might have affected the choice and use of anticoagulants. (3) In addition, some referenced studies targeted acute VTE without cancer. However, to the best of our knowledge, this clinical study is the first to report the efficacy of a single-drug approach with edoxaban for the treatment of non-acute CAT; hence, future studies can use these results to obtain evidence without bias.

## 5. Conclusions

Although this was not a randomized controlled study, we demonstrated that a single-drug approach with edoxaban, a drug that is largely unaffected by CYP3A4, is a potential treatment option for non-acute CAT. We believe that as this drug is unlikely to cause drug-drug interactions, its efficacy makes it a promising option for treating cancer in an outpatient setting.

## Figures and Tables

**Table 1:** Demographic and baseline characteristics of patients (*n* = 32).

Characteristics	Patients (*n* = 32)
Age—year (IQR)	68 (60.75–75.25)
Sex—no. (%)	
Male	14 (43.8)
Female	18 (56.3)
Body surface area—m^2^ (IQR)	1.52 (1.38–1.60)
Body weight—kg (IQR)	50.55 (45.28–57.05)
≤60 kg—no. (%)	25 (78.1)
>60 kg—no. (%)	7 (21.9)
Creatinine Clearance (Cockcroft-Gault Equation)—mL/min (IQR)	66.71 (54.10–85.84)
≤50 mL/min—no. (%)	7 (21.9)
>50 mL/min—no. (%)	25 (78.1)
The dose of edoxaban—no. (%)	
30 mg	25 (78.1)
60 mg	7 (21.9)
Type of thrombosis—no. (%)	
Pulmonary embolism	8 (25.0)
Proximal deep-venous thrombosis	9 (28.1)
Distal deep-venous thrombosis	21 (65.6)
Symptomatic venous thromboembolism	4 (12.5)
Type of diagnosis—no. (%)	
Symptomatic CAT diagnosed more than 14 days after onset	4 (12.5)
Asymptomatic CAT diagnosed by the D-dimer/CT approach	28 (87.5)
Chemotherapy target—no. (%)	
Primary advanced	22 (68.8)
Recurrence	7 (21.9)
Adjuvant	3 (9.4)
The number of chemotherapy lines—no. (%)	
0	3 (9.4)
1	23 (71.9)
2	5 (15.6)
3	0 (0.0)
4 or more	1 (3.1)
ECOG performance status—no. (%)	
0	18 (56.3)
1	12 (37.5)
2	2 (6.3)
3	0 (0.0)
4	0 (0.0)
Administration history of VEGF inhibitors—no. (%)	9 (28.1)
Onset during the administration of VEGF inhibitors—no. (%)	8 (25.0)
Follow-up period—day (IQR)	335.5 (245–390)

Abbreviations: IQR, interquartile range; ECOG, Eastern Cooperative Oncology Group; and VEGF, vascular endothelial growth factor.

**Table 2:** Clinical outcomes during the overall analysis period.

Endpoints	Patients (*n* = 32)
**Primary endpoint**	
Thrombus disappearance at the first evaluation—no. (%; 95% CI)	20 (62.5; 43.7–78.9)
**Secondary endpoints**	
Recurrent venous thromboembolism—no. (%; 95% CI)	2 (6.25; 0.8–20.8)
Recurrent pulmonary embolism—no. (%; 95% CI)	1 (3.13; 0.1–16.2)
Recurrent deep-vein thrombosis—no. (%; 95% CI)	2 (6.25; 0.8–20.8)
Major bleeding—no. (%; 95% CI)	2 (6.25; 0.8–20.8)
Fatal bleeding—no. (%; 95% CI)	0 (0.00; 0.0–8.9)
Median D-dimer levels at diagnosis—μg/mL (IQR)	5.0 (2.80–8.60)
Median D-dimer levels after 1 month—μg/mL (IQR)	1.1 (0.85–1.65)

Abbreviations: CI, confidence interval; IQR, interquartile range.
